# Microarrays for Bacterial Pathogens — Hope or Hype?

**DOI:** 10.1002/cfg.180

**Published:** 2002-08

**Authors:** Brendan W. Wren

**Affiliations:** Pathogen Molecular Biology and Biochemistry Unit Department of Infectious and Tropical Diseases London School of Hygiene and Tropical Medicine London WC1E 7HT UK


**Comparative and Functional Genomics**
*Comp Funct Genom* 2002; **3**: 330–332.
Published online in Wiley InterScience (www.interscience.wiley.com). **DOI:** 10.1002/cfg.180

**Conference Discussion**

### Microarrays for bacterial pathogens — hope or hype?

Brendan W. Wren*
*Department of Infectious and Tropical Diseases, London School of Hygiene and Tropical Medicine, London WC1E 7HT, UK*

**Correspondence to:
Brendan W. Wren, Pathogen Molecular Biology and Biochemistry Unit, Department of Infectious and Tropical Diseases, London School of Hygiene and Tropical Medicine, London WC1E 7HT, UK.
E-mail:
brendan.wren@lshtm.ac.uk*

Received: 31 May 2002
Accepted: 7 June 2002

Despite advances in the treatment of infectious disease, pathogenic bacteria still represent one of the most important threats to human health worldwide. Many infectious disease agents have never been controlled, or have re-emerged as global pathogens, while others pose a new threat. Media and scientific attention has focused on a range of problems, including the alarming spread of antibiotic resistance, the threat of bioterrorism, microbial contamination of food, the global resurgence of tuberculosis, and other emerging and re-emerging infections triggered by lifestyle, political, economic and ecological changes. It is also becoming increasingly clear that bacteria have a causative role in major diseases such as cancer (e.g. *Helicobacter pylori* and gastric cancer) and heart disease (e.g. *Chlamydia pneumoniae*). The need to gain an integrated and comprehensive understanding of the workings of our old bacterial adversaries is as great as ever. Do DNA microarrays provide the hope?

DNA microarrays are the current tool for high-throughput hybridization analysis. Generally they consist of amplified gene fragments representing individual genes that are robotically printed on suitably coated glass slides. There two principal experiments that can be performed with microarrays, hybridization of DNA (often referred to as genomotyping) or hybridization of mRNA (expression analysis). Much hype has surrounded the application and exploitation of DNA microarrays, particularly expression analysis, where it was hoped that it would be possible to monitor at a glance the temporal and spatial expression of all pathogen and host genes during infection. Not too long ago there were more reviews on the subject than original articles. So what is the reality?

### DNA hybridization to microarrays

The definitive information a medical microbiologist would want from a bacterium is its entire DNA sequence, as and when it is isolated. Currently, sequencing the genomes of bacterial pathogens on a routine basis is not possible, but hybridization of DNA from the test sample to a carefully designed microarray can be very informative. Single organism and even composite species microarrays have been designed for many sequenced bacteria. Data from these studies have

Copyright © 2002 John Wiley & Sons, Ltd.

spawned the research disciplines of comparative or evolutionary genomics.

Examples of the application of DNA microarrays include a retrospective study to address the issue of whether strains responsible for the current seventh cholera pandemic have genes encoding gain-of-function traits that may have displaced pre-existing classical *V. cholerae* strains (Dziejman *et al.*, 2002). A microarray consisting of >93% of the genes of the El Tor O1 N16961 strain was used to analyse a collection of nine strains of diverse global origin isolated between 1910 and 1992. Of the gene differences, two putative chromosomal islands (VSP-I and VSP-II) with a deviant G+C content were identified (Dziejman *et al.*, 2002). These genes may encode adaptive properties that allow these strains to withstand nutrient deprivation or stresses and thus survive more efficiently in aquatic environments than strains before the seventh pandemic. The genes identified by microarray analysis can now be deleted systematically to determine their potential role in human infection and in promoting the fitness of *V. cholerae* in environmental ecosystems.

Another retrospective study was used to determine the genome content of *Staphylococcus aureus* strains responsible for the toxic shock syndrome (TSS) epidemic of the 1970s. Of the *S. aureus* strains isolated from women with urogenital-associated TSS, about 90% have a distinct multilocus enzyme genotype, designated ET 234. Although the DNA microarray data confirmed that ET 234 strains are genetically related and have shared a common ancestor, it also revealed that these strains are not genetically identical and that the last ancestor has not been very recent in evolutionary time. It was concluded that the spate of TSS was caused by host factors (the use of a new superabsorbent tampon) rather than the rapid global dissemination of a hyperinvasive strain (Fitzgerald *et al.*, 2001).

Microarray studies have also been conducted where the bacterium of interest has not been sequenced, but has a close relative with a fully sequenced genome, e.g. *Wigglesworthia glossinidia*, a member of the *Enterobacteriaceae*, is an obligate endosymbiont of the tsetse fly, which relies on the bacterium for fertility and nutrition. Symbiotic associations with microorganisms are pivotal in many insects, but the functional role of obligate symbionts can be difficult to study, due to the problem of growing these organisms *in vitro*. The *W. glossinidia* genome is less than 770 kb, about one-sixth that of the related free-living bacterium *E. coli* (4.6 Mb). In order to gain an insight into the composition of the genome, *W. glossinidia* genomic DNA was hybridized to an *E. coli* DNA microarray, revealing 650 orthologous genes, corresponding to approximately 85% of the genome (Akman and Aksoy, 2001). Many of the genes retained in the *W. glossinidia* genome are involved in cell processes, DNA replication, transcription and translation. However, genes encoding transport proteins, chaperones, biosynthesis of co-factors and some amino acids were also identified in significant numbers, suggesting an important role for these proteins in the bacterium's symbiotic lifestyle. This is a good example of how a bacterial microarray can be used to obtain broad genome information for a closely related organism in the absence of complete genome sequence data.

### mRNA hybridization to microarrays

In contrast to the diverse range of comparative genomics studies using microarrays, there have been fewer reports of hybridizing bacterial mRNA to pathogen microarrays. This is partly due to the difficulties associated in purifying mRNA that is representative of the organism's response at the appropriate moment in time during *in vitro* or *in vivo* environmental stress. Methods for purifying bacterial mRNA in the presence of eukaryotic cells have not been forthcoming, the exception being the differential lysis method developed by Mangan and Butcher to isolate *Mycobacterium bovis* BCG mRNA after phagocytosis by macrophages (Li *et al.*, 2001). Many *in vitro* stresses that may mimic *in vivo* infection scenarios have been attempted, such as shifts in temperature, pH and oxidation, or through the addition of quorum sensing inducers, bile salts and antibiotics, but the interpretation and relevance of the data to the infection process is questionable. However, experiments using defined regulatory mutants, such as sigma factor or response regulator mutants (presented at this meeting), have clearly shown the potential for microarrays to decipher regulatory networks in pathogenic bacteria, which in turn should enable us to rip out the wiring diagram of bacteria to find out what makes them tick.

Copyright © 2002 John Wiley & Sons, Ltd.

### Future perspectives

All microarray analyses are limited by the genetic information on the array. However, as more genomes are sequenced and the capacity of arrays is increased further, information on arrays can be used to interrogate a given bacterial genome or mixture of genomes. Apart from bacterial mRNA isolation, another stumbling block to reaping the benefits of DNA microarrays is insufficient bioinformatic tools to analyse data. In the future, sustained improvements in software, computing speed and information storage will dramatically increase the scale of problems we tackle to understand the basic biology and evolution of the microbes. The development of a database of nucleotide differences among strains should allow the design of a universal microbial pathogen microarray that would have wide applications in studying the epidemiology, population genetics, molecular phylogeny and evolution of bacterial pathogens, as well as diagnostic applications. A 'lateral gene transfer' microarray consisting of genes from mobile elements such as pathogenicity islands, phage and plasmid sequences may have multiple applications, e.g. it could be used in active microbial surveillance as an early warning system to alert public health officials to the potential emergence of a more virulent pathogen. Currently, we have only just begun to scratch the surface in terms of the potential applications of DNA microarrays. The next few years promise to be a voyage of discovery in terms of developing our understanding of bacterial pathogens. There is no doubt that the knowledge garnered from these studies will be applied to well-designed intervention strategies to reduce the burden of infectious disease.

### Acknowledgements

I would like to thank the Wellcome Trust for the funding of the B$\mu$G@S facility.

### References

Akman L, Aksoy S. 2001. A novel application of gene arrays: *Escherichia coli* array provides insight into the biology of the obligate endosymbiont of tsetse flies. *Proc Natl Acad Sci USA* **98**(13): 7546–7551.

Dziejman M, Balon E, Boyd D, Fraser CM, Heidelberg JF, Mekalanos JJ. 2002. Comparative genomic analysis of *Vibrio cholerae*: genes that correlate with cholera endemic and pandemic disease. *Proc Natl Acad Sci USA* **99**(3): 1556–1561.

Fitzgerald JR, Sturdevant DE, Mackie SM, Gill SR, Musser JM. 2001. Evolutionary genomics of *Staphylococcus aureus*: insights into the origin of methicillin-resistant strains and the toxic shock syndrome epidemic. *Proc Natl Acad Sci USA* **98**(15): 8821–8826.

Li MS, Monahan IM, Waddell SJ, *et al.* 2001. cDNA-RNA subtractive hybridization reveals increased expression of mycocerosic acid synthase in intracellular *Mycobacterium bovis* BCG. *Microbiology* **147**(8): 2293–305.

Copyright © 2002 John Wiley & Sons, Ltd. 
